# Mutational Spectrum and Clinical Outcomes of Myelodysplastic/Myeloproliferative Neoplasms: A Single-Institution Study in Korea with Emphasis on *U2AF1*

**DOI:** 10.3390/jcm14197074

**Published:** 2025-10-07

**Authors:** Min-Seung Park, Dae-Ho Choi, Jun Ho Jang, Chul Won Jung, Hee-Jin Kim, Hyun-Young Kim

**Affiliations:** 1 Department of Laboratory Medicine, Kangbuk Samsung Hospital, Sungkyunkwan University School of Medicine, Seoul 03181, Republic of Korea; ms0222.park@gmail.com; 2 Division of Hematology-Oncology, Department of Medicine, Samsung Medical Center, Sungkyunkwan University School of Medicine, Seoul 06351, Republic of Korea; daeho619.choi@samsung.com (D.-H.C.); jh21.jang@samsung.com (J.H.J.); chulwon1.jung@samsung.com (C.W.J.); 3 Department of Laboratory Medicine and Genetics, Samsung Medical Center, Sungkyunkwan University School of Medicine, Seoul 06351, Republic of Korea; heejinkim@skku.edu

**Keywords:** myelodysplastic/myeloproliferative neoplasm, chronic myelomonocytic leukemia, *U2AF1*, next-generation sequencing, prognostic factor

## Abstract

**Background**: Myelodysplastic/myeloproliferative neoplasms (MDS/MPNs) are rare hematopoietic disorders that exhibit overlapping pathological and molecular features of both MDS and MPN. This study aimed to investigate the mutational profiles and prognostic implications of MDS/MPN subtypes in Korean patients. **Methods**: We retrospectively reviewed 53 patients with MDS/MPN who underwent bone marrow examination and next-generation sequencing panel testing. Overall survival was analyzed with 3-year censoring. The cohort included chronic myelomonocytic leukemia (CMML; *N* = 30); MDS/MPN with neutrophilia (*N* = 6); MDS/MPN with *SF3B1* mutation and thrombocytosis (*N =* 4); and MDS/MPN, not otherwise specified (MDS/MPN-NOS; *N =* 13). **Results**: The most frequently mutated gene was *ASXL1* (52.8%), followed by *TET2* (39.6%) and *U2AF1* (18.9%), in total MDS/MPN. *U2AF1* mutations were particularly frequent in myelodysplastic CMML (33.3%) and MDS/MPN-NOS (30.8%). In CMML, *ASXL1* and *TET2* mutations were associated with a trend toward better prognosis compared with wild-type (HR 0.21, *p* = 0.052; HR 0.25, *p* = 0.057, respectively), while *U2AF1* mutations were associated with adverse prognosis in univariate analysis with borderline significance (HR 12.20, *p* = 0.050). Clinical/Molecular CMML-Specific Prognostic Scoring System and Mayo Molecular Model showed stepwise survival patterns across risk groups without statistical significance. In univariate analysis, transfusion dependency was associated with poor prognosis (HR 7.78, *p* = 0.013). **Conclusions**: This study provides the first single-institution analysis in Korean patients with MDS/MPN and identified *U2AF1* mutations as a potentially significant prognostic factor, enhancing the molecular understanding of MDS/MPN.

## 1. Introduction

Myelodysplastic/myeloproliferative neoplasms (MDS/MPNs) represent a rare group of clonal hematopoietic disorders that exhibit overlapping pathologic and molecular features of both MDS and MPN [1,2]. The World Health Organization (WHO) 5th edition classification recognizes four distinct subtypes within this category: chronic myelomonocytic leukemia (CMML); MDS/MPN with neutrophilia (MDS/MPN-N); MDS/MPN with *SF3B1* mutation and thrombocytosis (MDS/MPN-*SF3B1*-T); and MDS/MPN, not otherwise specified (MDS/MPN-NOS) [3]. While these entities share the common characteristic of combined cytopenias and proliferative features on peripheral blood, they can be distinguished by specific hematologic and molecular criteria. CMML is defined by persistent peripheral blood monocytosis (absolute count ≥ 0.5 × 10^9^/L and relative count ≥ 10% of white blood cells [WBCs]), with additional diagnostic requirements varying based on the absolute monocyte count; MDS/MPN-N is characterized by neutrophilia with dysplasia and ≥ 10% circulating immature myeloid cells; and MDS/MPN-*SF3B1*-T presents with thrombocytosis in conjunction with *SF3B1* mutation and associated dyserythropoiesis, frequently manifesting with ring sideroblasts [4].

CMML, the most common subtype among MDS/MPNs, is further subclassified based on WBC count into myelodysplastic CMML (MD-CMML, WBC count < 13 × 10^9^/L) and myeloproliferative CMML (MP-CMML, WBC count ≥ 13 × 10^9^/L) [5]. These subtypes demonstrate distinct clinicopathological characteristics with differences in mutational distribution and prognosis [6]. The molecular landscape of CMML is characterized by recurrent mutations affecting three major pathways: epigenetic regulation of transcription and histone modification (e.g., *TET2* and *ASXL1*), RNA splicing factors (e.g., *SRSF2*, *U2AF1*, and *SF3B1*), and signal transduction pathways (e.g., *KRAS*, *NRAS*, and *CBL*) [5]. Notably, mutations in epigenetic and splicing genes are more frequently associated with MD-CMML, while alterations in cellular signaling pathways show stronger association with MP-CMML [7]. *U2AF1* mutations occur predominantly at two hotspots (S34 and Q157) with distinct biological effects, particularly S34 mutations showing growth-inhibitory properties that may contribute to their enrichment in the more dysplastic subtype [8,9]. MDS/MPN-N and MDS/MPN-*SF3B1*-T share similar overall mutational profiles with CMML, though specific mutations show strong disease associations: *SETBP1* and *ETNK1* mutations are particularly enriched in MDS/MPN-N, while *SF3B1* mutations are, by definition, strongly associated with MDS/MPN-*SF3B1*-T [10,11].

Risk assessment in MDS/MPN has been most extensively studied in CMML, with several prognostic scoring systems proposed [6]. Among these, the Clinical/Molecular CMML-Specific Prognostic Scoring System (CPSS-Mol) and the Mayo Molecular Model (MMM) have gained widespread acceptance [6]. The CPSS-Mol incorporates clinical parameters including red blood cell (RBC) transfusion dependency, WBC count, hemoglobin (Hb) level, platelet count, bone marrow (BM) blast percentage, and cytogenetic risk groups, along with molecular markers such as *ASXL1*, *NRAS*, *RUNX1*, and *SETBP1* mutations [12]. In contrast, the MMM provides a more streamlined approach, excluding RBC transfusion dependency and cytogenetic abnormalities while incorporating only *ASXL1* mutation status among molecular markers, alongside absolute monocyte count, Hb level, platelet count, and presence of circulating immature myeloid cells [13].

Given the rarity of MDS/MPNs and the recent adoption of next-generation sequencing (NGS)-based multigene panel testing in clinical practice, data on the molecular profiles of these disorders in Korean patients remain limited. This study aimed to comprehensively characterize the mutational landscape of MDS/MPN subtypes in Korean patients and to assess the applicability of molecular-based prognostic systems.

## 2. Materials and Methods

### 2.1. Study Participants

We retrospectively reviewed 53 patients diagnosed with MDS/MPN who underwent NGS gene panel testing between March 2019 and June 2025, including 15 patients with MD-CMML (WBC count < 13 × 10^9^/L; 14 with CMML-1 and one with CMML-2), 15 with MP-CMML (WBC count ≥ 13 × 10^9^/L; 12 with CMML-1 and 3 with CMML-2), 6 with MDS/MPN-N, 4 with MDS/MPN-*SF3B1*-T, and 13 with MDS/MPN-NOS. All BM diagnoses were re-evaluated according to the WHO 5th edition classification criteria [3], and diagnoses were revised when necessary. Clinical and laboratory data were collected from electronic medical records, including sex, age, complete blood cell count, BM examination results, conventional cytogenetics, fluorescence in situ hybridization (FISH), presence of splenomegaly, RBC transfusion dependency at diagnosis (defined as requiring at least one unit of RBC every 8 weeks over a 16 weeks period) [14], and treatment regimens. This study was approved by the Institutional Review Board of Samsung Medical Center with a waiver of informed consent (SMC 2025-08-126).

### 2.2. Next-Generation Sequencing for Myeloid Neoplasm-Related Genes

Genomic DNA was extracted from BM aspirates or peripheral blood samples using the Wizard Genomic DNA Purification Kit (Promega, Madison, WI, USA). Library preparation was conducted using IDT xGen pre-designed/custom probes (Integrated DNA Technologies, Coralville, IA, USA). Following library preparation, targeted sequencing was performed on the NextSeq 550Dx platform (Illumina, San Diego, CA, USA). The sequencing panel included 37 myeloid neoplasm-associated genes (*ABL1*, *ASXL1*, *BCOR*, *BRAF*, *CALR*, *CBL*, *CEBPA*, *CSF3R*, *DNMT3A*, *ETV6*, *EZH2*, *FLT3*, *GATA2*, *HRAS*, *IDH1*, *IDH2*, *JAK2*, *KIT*, *KRAS*, *MPL*, *NF1*, *NPM1*, *NRAS*, *PRPF8*, *PTPN11*, *RB1*, *RUNX1*, *SETBP1*, *SF3B1*, *SH2B3*, *SRSF2*, *STAG2*, *TET2*, *TP53*, *U2AF1*, *WT1*, and *ZRSR2*) [15,16,17]. Sequencing reads were aligned to the GRCh37/hg19 reference using BWA-MEM, processed with Picard, and variants were called with GATK (HaplotypeCaller, Mutect2), Pisces, and Pindel. Variants were filtered with custom quality control scripts and annotated using snpEff with population databases. Target coverage depth was 1000×, with variant calling performed using threshold of ≥2.0% variant allele frequency (VAF) or ≥20 supporting reads. NGS analysis was performed using tumor samples only, without matched normal tissue samples. Identified variants were interpreted according to the 2017 AMP/ASCO/CAP somatic tier guidelines and 2022 ClinGen/CGC/VICC oncogenicity guidelines [18,19], and (likely) oncogenic variants classified as tier 1 or 2 were included in this study.

### 2.3. Statistical Analysis

Overall survival (OS) was defined as the time from initial diagnosis to death from any cause or last follow-up, with patients censored at 3 years for analysis due to heterogeneity in follow-up duration. Kaplan–Meier curves with log-rank tests were used to compare survival between MDS/MPN subtypes and validate risk stratification (CPSS-Mol and MMM scoring systems) [12,13]. The discriminatory ability of prognostic scoring systems was assessed using concordance index (C-index) with 95% confidence intervals (CIs). For evaluation of individual prognostic factors, univariate Cox proportional regression was performed to calculate hazard ratios (HRs) and 95% CIs. For multivariate analysis, a pre-specified model including age, CMML subtype (MD vs. MP), high-risk cytogenetics, and mutational status of *ASXL1* and *U2AF1* was analyzed using Firth-penalized Cox regression due to the limited number of events. All statistical analyses and data visualization were performed using R software version 4.5.1 (R Foundation for Statistical Computing, Vienna, Austria), and a *p*-value < 0.05 was considered statistically significant.

## 3. Results

### 3.1. Patients’ Characteristics

Clinical and laboratory characteristics of the 53 patients with MDS/MPN and their subtypes are summarized in Table 1. The cohort was predominantly male (69.8%) with a median age of 68 years (range, 37–87). At diagnosis, median WBC count was 18.57 × 10^9^/L (range, 2.97–136.82), Hb 8.2 g/dL (5.4–15.5), and platelet count 107 × 10^9^/L (6–1343). Morphologic dysplasia was most frequently observed in the megakaryocytic lineage (84.9%), followed by granulocytic (75.5%) and erythroid lineages (54.7%). Grade 2 or higher myelofibrosis was present in 26.4% of patients. Cytogenetic analysis, including conventional karyotyping and FISH, revealed monosomy 7/del(7q) and trisomy 8 as the most frequent abnormalities, each occurring in 11.3% of patients. Patients harbored a median of 3 mutated genes (range, 0–7), and *ASXL1* and *TET2* co-mutations were observed in 22.6%. Splenomegaly was observed in 17.0% of patients at diagnosis. RBC transfusion dependency was present in 37.7% of patients at initial diagnosis, and 9.4% underwent allogeneic hematopoietic stem cell transplantation during the follow-up period. Disease progression or relapse within 3 years of diagnosis occurred in 15.1% of patients, and 26.4% died during a median follow-up of 1.16 years (interquartile range, 0.19–2.85 years; reverse Kaplan–Meier method).

### 3.2. Mutational Landscape Across MDS/MPN Subtypes

**Figure 1:**
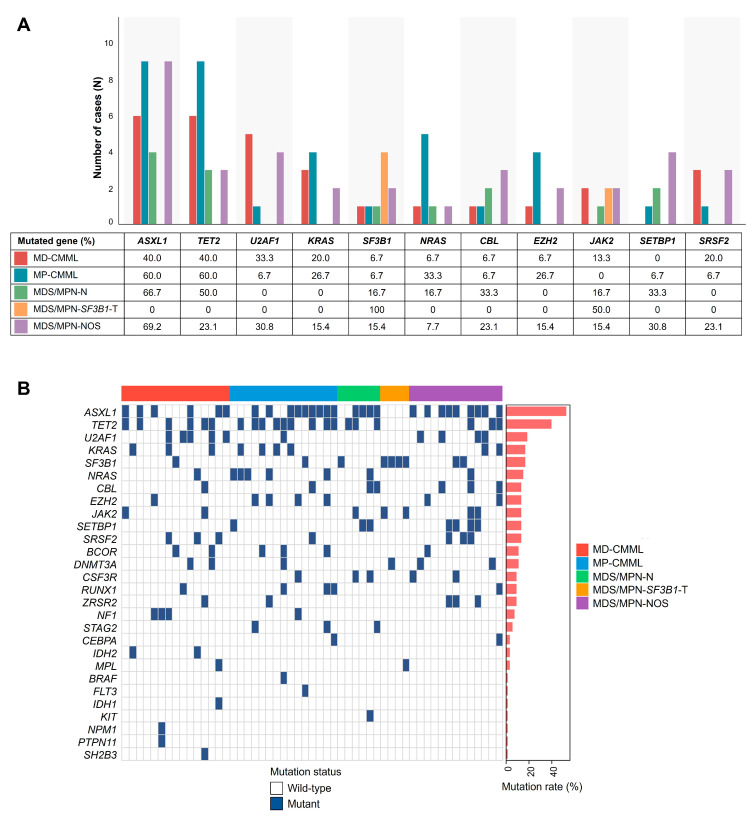
(**A**) Distribution of major mutated genes across MDS/MPN subtypes. (**B**) Oncoprint of genetic mutations in MDS/MPN patients by disease subtype.

*ASXL1* and *TET2* were the most frequently mutated genes across MDS/MPN subtypes, with distinct patterns in each subtype (Figure 1A and Supplemental Table S1). Overall, *ASXL1* mutations occurred in 52.8% of patients, followed by *TET2* (39.6%), *U2AF1* (18.9%), *KRAS* (17.0%), *SF3B1* (17.0%), and *NRAS* (15.1%). In MD-CMML, *ASXL1* and *TET2* mutations were the most common (40.0% each), followed by *U2AF1* (33.3%), *KRAS*, *NF1*, and *SRSF2* (20.0% each). In MP-CMML, *ASXL1* and *TET2* mutations were also the most common (60.0% each), followed by *NRAS* (33.3%), *EZH2*, and *KRAS* (26.7% each), while *SRSF2* mutations were relatively rare (6.7%). In MDS/MPN-N, *ASXL1* (66.7%) and *TET2* (50.0%) mutations were the most frequent, followed by *CBL*, *CSF3R*, and *SETBP1* (33.3% each). Patients with MDS/MPN-*SF3B1*-T uniformly harbored *SF3B1* mutations (100%) and frequently exhibited *JAK2* mutations (50.0%). In MDS/MPN-NOS, *ASXL1* was the most prevalent (69.2%), followed by *SETBP1* and *U2AF1* (30.8% each). Detailed oncoprint visualization by disease subtype is presented in Figure 1B.

Among *U2AF1* mutations (*N =* 10), S34 hotspot mutations were predominant (60.0%), including S34F (*N =* 5) and S34Y (*N =* 1), while Q157P mutations occurred in 4 patients (40.0%) (Supplemental Figure S1). The VAFs ranged from 9.8% to 52.3% (median, 34.0%). *U2AF1* mutations showed differential distribution across subtypes, being more frequent in MD-CMML (5/15, 33.3%) compared to MP-CMML (1/15, 6.7%), with S34 mutations predominating in MD-CMML (4/5, 80.0%).

### 3.3. Survival and Prognostic Factors in the MDS/MPN Cohort

#### 3.3.1. Survival Analysis by MDS/MPN Subtypes

**Figure 2:**
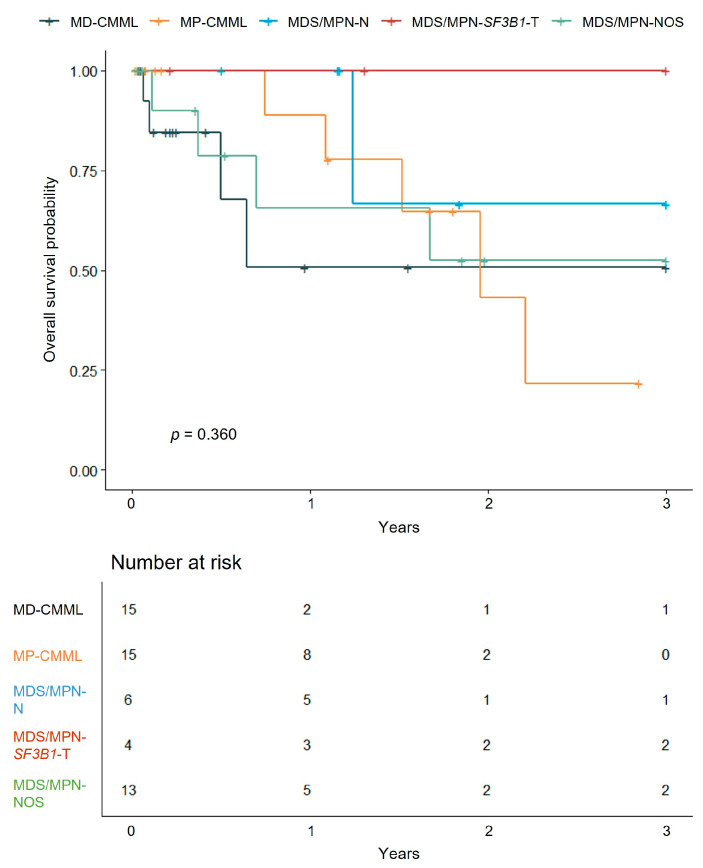
Kaplan–Meier curves for overall survival by MDS/MPN subtypes. Event counts (deaths/total patients) are as follows: MD-CMML (4/15), MP-CMML (5/15), MDS/MPN-N (1/6), MDS/MPN-*SF3B1*-T (0/4), and MDS/MPN-NOS (4/13).

No significant survival differences were observed across MDS/MPN subtypes (*p* = 0.360) (Figure 2), likely due to small sample sizes and limited follow-up. Estimated OS probabilities at the last follow-up within 3 years were 50.8% for MD-CMML (3.0 years; 95% CI: 23.9–100%), 21.6% for MP-CMML (2.85 years; 95% CI: 4.0–100%), 66.7% for MDS/MPN-N (3.0 years; 95% CI: 30.0–100%), 100% for MDS/MPN-*SF3B1*-T (3.0 years; 95% CI: not estimable), and 52.5% for MDS/MPN-NOS (3.0 years; 95% CI: 27.2–100%). Although MP-CMML showed a trend toward inferior survival, pairwise comparisons between subtypes did not reach statistical significance (all *p* > 0.05).

#### 3.3.2. Prognostic Impact of Gene Mutations in MDS/MPN

**Figure 3:**
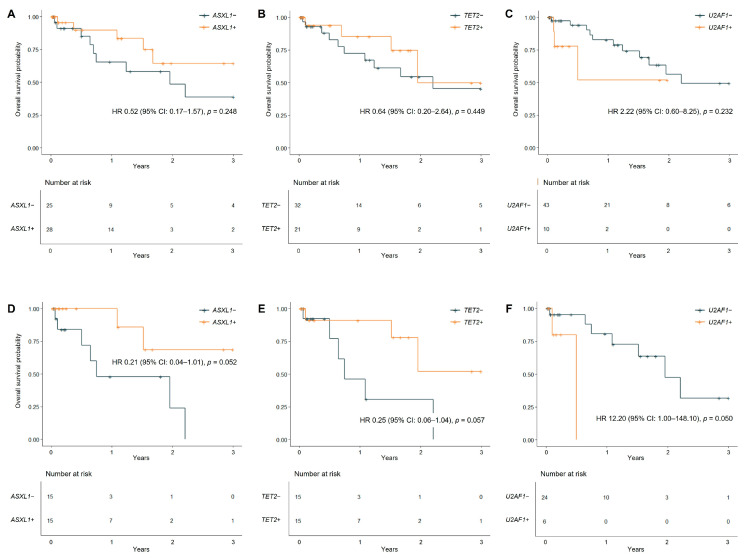
Kaplan–Meier plots for overall survival according to *ASXL1*, *TET2*, and *U2AF1* mutations in the entire MDS/MPN cohort ((**A**–**C**), *N =* 53) and the CMML subtype ((**D**–**F**), *N =* 30). In total MDS/MPN cohort (**A**–**C**), event counts (deaths/total patients) are *ASXL1* wild-type (9/25), *ASXL1* mutant (5/28), *TET2* wild-type (10/32), *TET2* mutant (4/21), *U2AF1* wild-type (11/43), and *U2AF1* mutant (3/10). In *CMML* cohort (**D**–**F**), *ASXL1* wild-type (7/15), *ASXL1* mutant (2/15), *TET2* wild-type (6/15), *TET2* mutant (3/15), *U2AF1* wild-type (7/24), and *U2AF1* mutant (2/6).

Mutations in *ASXL1*, *TET2*, and *U2AF1* showed varying prognostic trends in the overall MDS/MPN cohort. Patients with *ASXL1* and *TET2* wild-type showed a trend toward inferior OS compared to those with mutations, while *U2AF1* mutations showed a trend toward adverse prognosis (Figure 3A–C). However, these trends did not reach statistical significance (all *p* > 0.05).

### 3.4. Survival and Prognostic Factors in CMML

#### 3.4.1. Prognostic Impact of Gene Mutations

In CMML patients, *U2AF1* mutations showed borderline significance with worse survival (HR 12.2, 95% CI: 1.0–148.1, *p* = 0.050), while *ASXL1* and *TET2* mutations showed favorable trends (*p* = 0.052 and *p* = 0.057, respectively) (Figure 3D–F).

#### 3.4.2. Survival Analysis by Prognostic Scoring Systems

**Figure 4:**
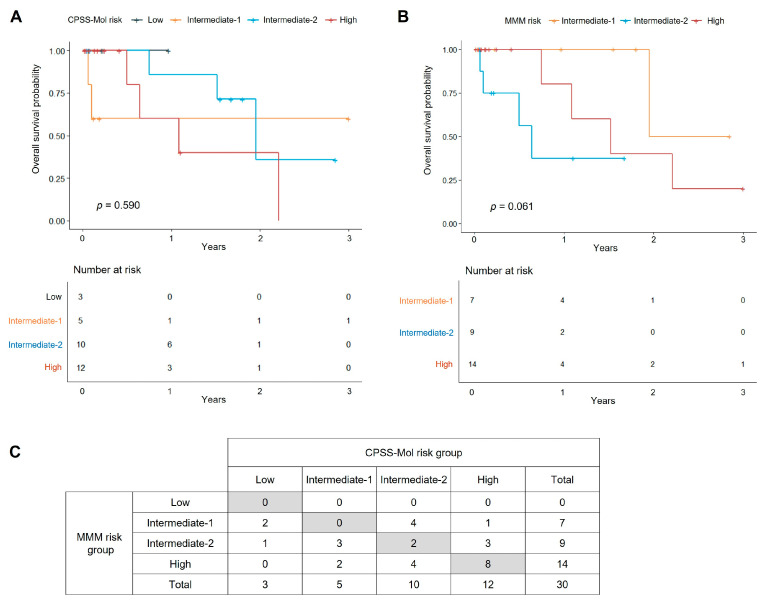
Overall survival of CMML patients stratified by (**A**) CPSS-Mol and (**B**) MMM risk categories, and (**C**) concordance between the two models. Gray-shaded areas indicate identical risk category assignments between CPSS-Mol and MMM.

Survival in CMML patients varied by prognostic scoring systems, though differences were not statistically significant (Figure 4). Using CPSS-Mol, OS estimates at the last follow-up within 3 years were 100% for low risk (0.97 years; 95% CI not estimable), 60.0% for intermediate-1 (3.0 years; 95% CI 29.3–100%), 35.7% for intermediate-2 (2.85 years; 95% CI 8.3–100%), and 0% for high risk (2.21 years; 95% CI not estimable). By MMM categories, OS estimates were 50.0% for intermediate-1 (2.85 years; 95% CI 12.5–100%), 37.5% for intermediate-2 (1.67 years; 95% CI 13.0–100%), and 20.0% for high risk (3.0 years; 95% CI 3.5–100%). Higher-risk groups showed trends toward inferior survival, but differences were not statistically significant (*p* = 0.590 for CPSS-Mol and *p* = 0.061 for MMM; all pairwise *p* > 0.05). The C- index was 0.510 (95% CI: 0.039–0.981) for CPSS-Mol and 0.466 (95% CI: 0.105–0.828) for MMM.

#### 3.4.3. Clinical and Molecular Prognostic Variables

Univariate Cox regression identified transfusion dependency as a significant adverse prognostic factor for OS in CMML (HR 7.78, 95% CI: 1.55–39.23, *p* = 0.013) (Supplemental Table S2 and Figure S2). Low platelet count, high-risk cytogenetics, absence of *ASXL1* mutation, and presence of *U2AF1* mutation showed borderline associations with poor prognosis, but none retained independent significance in multivariate analysis.

## 4. Discussion

Given the clinical and biological heterogeneity of MDS/MPN, comprehensive molecular characterization is critical to improve prognostication and therapeutic strategies. Accordingly, following the implementation of NGS in routine clinical practice, we conducted a retrospective analysis to characterize the mutational profiles and clinical outcomes of Korean patients with MDS/MPN. However, the single-institution design and limited sample sizes of this study necessitate careful interpretation of subtype-specific findings.

In CMML, *ASXL1* and *TET2* were the most frequently mutated genes, observed in 40.0% of MD-CMML and 60.0% of MP-CMML patients. These frequencies are consistent with previous studies reporting *ASXL1* mutations in 26–45% and *TET2* mutations in 46–59% of CMML cases [20,21]. In contrast, *U2AF1* mutations were more frequent in our study (20.0% in CMML) compared to the 5–10% typically reported [6,22]. Conversely, *SRSF2* mutations were less common (13.3% in CMML) than the 25–47% previously reported [23]. Notably, previous studies in Chinese cohorts have also shown variability, with one reporting a *U2AF1* mutation frequency of 4.3% [24] and another reporting 23%, the latter being associated with an absolute monocyte count (AMC) < 1 × 10^9^/L [25]. Similarly, in our study, *U2AF1* mutations were more frequent in MD-CMML (33.3%) compared with MP-CMML (6.7%), suggesting that the growth-inhibitory effects of *U2AF1* mutations align with the dysplastic phenotype [8]. In our study, both S34 (S34F/S34Y) and Q157P *U2AF1* mutations were identified, with S34 being slightly more frequent. The higher *U2AF1* mutation frequency in our cohort may reflect ethnic predilection in East Asian populations, while the mutually exclusive relationship between *U2AF1* and *SRSF2* splicing factor mutations may explain the relatively lower *SRSF2* mutation frequency [9].

In MDS/MPN-N, *ASXL1* (66.7%) and *TET2* (50.0%) were also the most commonly mutated genes, with frequencies at the upper end of the reported ranges of 22–86% and 8–40%, respectively [26]. *SETBP1* (33.3%) and *CBL* (33.3%) were also recurrent. However, *ETNK1* mutations (typically 7–16% [26]) were not evaluated as this gene was not included in our sequencing panel. In MDS/MPN-*SF3B1*-T, *SF3B1* was universal (100%), with *JAK2* (50.0%) and *MPL* (25.0%) co-mutations comparable to published frequencies, whereas *TET2*, *DNMT3A*, *ASXL1* (15–30% in previous studies [11,27]) was absent. Given the small sample sizes of MDS/MPN-N and MDS/MPN-*SF3B1*-T, these frequencies may not accurately reflect the true distribution, and further studies with larger cohorts will be needed to confirm the overall mutational landscape. In MDS/MPN-NOS, *ASXL1* (69.2%), *U2AF1* (30.8%), and *SETBP1* (30.8%) occurred at higher frequencies than those reported in previous studies (21–55% [28,29], 11–18% [29,30], and 10–12% [29,30], respectively). These findings further support the observation of a relatively higher prevalence of *ASXL1* and *U2AF1* in Korean patients with MDS/MPN.

In the prognostic analysis by mutational status, statistical significance was not reached, but patients with *ASXL1* wild-type showed a trend toward poorer outcomes compared with those harboring *ASXL1* mutations. *ASXL1* encodes a chromatin regulator and has been consistently associated with adverse prognosis across myeloid neoplasms including AML, MDS, MPN, and MDS/MPN [31]. Our contradictory findings should be interpreted carefully, as they may be attributed to the skewed distribution of enrolled patients, with a large proportion classified into intermediate-2 and high risk groups (73.3% and 76.7% by CPSS-Mol and MMM, respectively), potential cohort bias from our single-institution design, and confounding effects of other concurrent mutations or clinical risk factors. *TET2* has been reported to exert a protective effect in CMML [32], and in our study, patients with *TET2* mutations demonstrated a tendency toward better outcomes. *U2AF1*, which is a spliceosome component involved in 3′ splice site recognition and has been associated with poor outcomes in previous studies [33], was the only mutation that reached borderline statistical significance (*p* = 0.050). Importantly, while the prognostic effect of *ASXL1* appeared diminished in advanced risk groups, *U2AF1* retained prognostic relevance. Considering the relatively high frequency and potential clinical utility of *U2AF1* in East Asian cohorts, our findings suggest that *U2AF1* may warrant consideration in future risk stratification models. However, in multivariate analysis, neither *U2AF1* mutations nor other variables included in established risk stratification systems achieved statistical significance, which may be attributed to the limited sample size of 30 CMML patients in our cohort. The loss of statistical significance in multivariate models likely reflects insufficient statistical power to detect independent prognostic effects when multiple variables are analyzed simultaneously. Therefore, larger multicenter studies with adequate sample sizes will be essential to validate the independent prognostic value of *U2AF1* mutations and to determine their potential incorporation into refined risk stratification models for CMML patients.

In the risk stratification analysis of CMML, both the CPSS-Mol and MMM demonstrated stepwise survival patterns across risk groups, but neither achieved statistical significance. C-index analysis further revealed poor discriminatory ability, with values approaching or below random prediction. The limited discriminatory performance is likely due to the small sample size and the low number of events, which reduced statistical power. Additionally, while these Western-derived scoring systems have demonstrated robust performance in larger international cohorts, their applicability to Korean patients may be influenced by population-specific genetic or clinical factors. These findings underscore the importance of validation in larger Korean cohorts to ascertain whether the observed limitations arise from sample size constraints or reflect true biological differences.

This study has several limitations. First, the small sample sizes in MDS/MPN subtypes other than CMML reduced the statistical power and limited the strength of subtype-specific conclusions. This constraint is further highlighted by unexpected findings such as the contradictory prognostic impact of *ASXL1* mutations and the lack of statistical significance in multivariate analysis. Second, our NGS panel did not include some diagnostically important genes such as *ETNK1*, which is frequently mutated in MDS/MPN-N [11]. Third, the analysis did not account for hematopoietic stem cell transplantation as a time-dependent covariate, which may have affected the survival estimates.

In conclusion, this study represents the first single-institution analysis of mutational and prognostic features in Korean patients with MDS/MPN, providing important insights into the mutational spectrum and its clinical implications. Despite the limitations of sample size, our findings suggest that *U2AF1* may play a particularly important role in Korean patients, both in terms of prevalence and prognostic impact. Further validation in larger, multi-institutional East Asian cohorts is essential to confirm the generalizability of these observations.

## Figures and Tables

**Table 1:** Clinical and laboratory characteristics of patients with MDS/MPN.

	Total MDS/MPN (*N =* 53)	MD-CMML (*N =* 15)	MP-CMML (*N =* 15)	MDS/MPN-N (*N =* 6)	MDS/MPN-*SF3B1*-T (*N =* 4)	MDS/MPN-NOS (*N* = 13)
Demographics						
Male	37 (69.8%)	10 (66.7%)	9 (60.0%)	5 (83.3%)	3 (75.0%)	10 (76.9%)
Age, years (range)	68 (37–87)	69 (41–79)	68 (37–85)	68 (41–85)	74 (65–82)	65 (40–87)
Laboratory findings						
WBC count (×10^9^/L)	18.57 (2.97–136.82)	6.94 (2.97–12.14)	27.07 (13.98–70.26)	40.92 (22.30–136.82)	4.80 (3.99–6.41)	19.53 (4.03–71.86)
Hb (g/dL)	8.2 (5.4–15.5)	8.7 (6.0–15.5)	8.1 (5.8–11.2)	9.4 (7.1–12.0)	8.4 (5.4–9.2)	8.1 (6.6–12.1)
PLT count (×10^9^/L)	107 (6–1343)	88 (11–625)	93 (6–347)	133 (29–480)	512 (434–721)	107 (11–1343)
Blast (%)	0 (0–8)	0 (0–4)	1 (0–7)	2 (0–3)	0 (0–0)	1 (0–8)
Monocyte (×10^9^/L)	2.31 (0.20–13.86)	2.03 (0.78–3.98)	6.38 (2.31–13.86)	2.22 (1.34–4.16)	0.42 (0.35–0.60)	0.63 (0.20–6.47)
Bone marrow findings						
Erythroid dysplasia	29 (54.7%)	9 (60.0%)	8 (53.3%)	4 (66.7%)	4 (100%)	5 (38.5%)
Granulocytic dysplasia	40 (75.5%)	12 (80.0%)	12 (80.0%)	6 (100%)	0 (0%)	10 (76.9%)
Megakaryocytic dysplasia	45 (84.9%)	14 (93.3%)	14 (93.3%)	6 (100%)	2 (50.0%)	9 (69.2%)
Myelofibrosis (grade ≥ 2)	14 (26.4%)	4 (26.7%)	4 (26.7%)	1 (16.7%)	1 (25.0%)	4 (30.8%)
Cytogenetic study						
-7 or del(7q)	6 (11.3%)	4 (26.7%)	1 (6.7%)	0 (0%)	0 (0%)	1 (7.7%)
Trisomy 8	6 (11.3%)	2 (13.3%)	2 (13.3%)	0 (0%)	0 (0%)	2 (15.4%)
17p loss	1 (1.9%)	0 (0%)	1 (6.7%)	0 (0%)	0 (0%)	0 (0%)
−20 or del(20q)	2 (3.8%)	0 (0%)	1 (6.7%)	0 (0%)	0 (0%)	1 (7.7%)
Y loss	1 (1.9%)	1 (6.7%)	0 (0%)	0 (0%)	0 (0%)	0 (0%)
Complex karyotype	5 (9.4%)	3 (20.0%)	0 (0%)	0 (0%)	0 (0%)	2 (15.4%)
NGS study						
Number of mutated gene	3 (0–7)	3 (0–5)	3 (1–7)	3 (1–6)	2 (1–3)	3 (0–7)
*ASXL1* & *TET2* co-mutation	12 (22.6%)	3 (20.0%)	5 (33.3%)	2 (33.3%)	0 (0%)	2 (15.4%)
Splenomegaly	9 (17.0%)	3 (20.0%)	3 (20.0%)	2 (33.3%)	0 (0%)	1 (7.7%)
Transfusion dependency	20 (37.7%)	7 (46.7%)	5 (33.3%)	1 (16.7%)	1 (25.0%)	6 (46.2%)
Treatment regimen						
HMA	8 (15.1%)	2 (13.3%)	4 (26.7%)	1 (16.7%)	0 (0%)	1 (7.7%)
HSCT	5 (9.4%)	0 (0%)	1 (6.7%)	3 (50.0%)	0 (0%)	1 (7.7%)
3-year outcome						
Progression	8 (15.1%)	1 (6.7%)	5 (33.3%)	1 (16.7%)	0 (0%)	1 (7.7%)
Death	14 (26.4%)	4 (26.7%)	5 (33.3%)	1 (16.7%)	0 (0%)	4 (30.8%)

Abbreviations: MDS/MPN, myelodysplastic/myeloproliferative neoplasm; CMML, chronic myelomonocytic leukemia; MDS/MPN-N, MDS/MPN with neutrophilia; MDS/MPN-*SF3B1*-T, MDS/MPN with *SF3B1* mutation and thrombocytosis; MDS/MPN-NOS, MDS/MPN not otherwise specified; WBC, white blood cell; Hb, hemoglobin; PLT, platelet; NGS, next-generation sequencing; HMA, hypomethylating agents; HSCT, hematopoietic stem cell transplantation; PFS, progression-free survival; OS, overall survival.

## Data Availability

All data relevant to this study have been included in the article or uploaded as Supplementary Information. The additional data presented in this study are available upon request from the corresponding author.

## Supplementary Materials

The following supporting information can be downloaded at: https://www.mdpi.com/article/10.3390/jcm14197074/s1, Table S1. Distribution of total mutated genes across MDS/MPN subtypes; Table S2. Univariate and multivariate Cox regression analysis for overall survival in patients with CMML; Figure S1. Distribution of variant allele frequencies of *U2AF1* mutations in patients with MDS/MPN, stratified by mutation hotspot (S34 vs. Q157); Figure S2. Forest plot of hazard ratios for 3-year overall survival in patients with CMML.
