# Supplementary material for: Mutational Spectrum and Clinical Outcomes of Myelodysplastic/Myeloproliferative Neoplasms: A Single-Institution Study in Korea with Emphasis on U2AF1
Source: J Clin Med. 2025 Oct 7;14(19):7074. doi: 10.3390/jcm14197074 (PMC12524835; doi:10.3390/jcm14197074)

Table S1. Distribution of total mutated genes across MDS/MPN subtypes.

Gene	Total MDS/MPN (N = 53)	MD-CMML (N = 15)	MP-CMML (N = 15)	MDS/MPN-N (N = 6)	MDS/MPN-*SF3B1*-T (N = 4)	MDS/MPN-NOS (N = 13)
ASXL1	28 (52.8%)	6 (40.0%)	9 (60.0%)	4 (66.7%)	0 (0%)	9 (69.2%)
TET2	21 (39.6%)	6 (40.0%)	9 (60.0%)	3 (50.0%)	0 (0%)	3 (23.1%)
U2AF1	10 (18.9%)	5 (33.3%)	1 (6.7%)	0 (0%)	0 (0%)	4 (30.8%)
KRAS	9 (17.0%)	3 (20.0%)	4 (26.7%)	0 (0%)	0 (0%)	2 (15.4%)
SF3B1	9 (17.0%)	1 (6.7%)	1 (6.7%)	1 (16.7%)	4 (100%)	2 (15.4%)
NRAS	8 (15.1%)	1 (6.7%)	5 (33.3%)	1 (16.7%)	0 (0%)	1 (7.7%)
CBL	7 (13.2%)	1 (6.7%)	1 (6.7%)	2 (33.3%)	0 (0%)	3 (23.1%)
EZH2	7 (13.2%)	1 (6.7%)	4 (26.7%)	0 (0%)	0 (0%)	2 (15.4%)
JAK2	7 (13.2%)	2 (13.3%)	0 (0%)	1 (16.7%)	2 (50.0%)	2 (15.4%)
SETBP1	7 (13.2%)	0 (0%)	1 (6.7%)	2 (33.3%)	0 (0%)	4 (30.8%)
SRSF2	7 (13.2%)	3 (20.0%)	1 (6.7%)	0 (0%)	0 (0%)	3 (23.1%)
BCOR	6 (11.3%)	2 (13.3%)	3 (20%)	0 (0%)	0 (0%)	1 (7.7%)
DNMT3A	6 (11.3%)	2 (13.3%)	1 (6.7%)	0 (0%)	1 (25.0%)	2 (15.4%)
CSF3R	5 (9.4%)	0 (0%)	1 (6.7%)	2 (33.3%)	0 (0%)	2 (15.4%)
RUNX1	5 (9.4%)	1 (6.7%)	3 (20.0%)	0 (0%)	0 (0%)	1 (7.7%)
ZRSR2	5 (9.4%)	1 (6.7%)	1 (6.7%)	0 (0%)	0 (0%)	3 (23.1%)
NF1	4 (7.5%)	3 (20.0%)	1 (6.7%)	0 (0%)	0 (0%)	0 (0%)
STAG2	3 (5.7%)	0 (0%)	2 (13.3%)	1 (16.7%)	0 (0%)	0 (0%)
CEBPA	2 (3.8%)	0 (0%)	1 (6.7%)	0 (0%)	0 (0%)	1 (7.7%)
IDH2	2 (3.8%)	2 (13.3%)	0 (0%)	0 (0%)	0 (0%)	0 (0%)
MPL	2 (3.8%)	1 (6.7%)	0 (0%)	0 (0%)	1 (25.0%)	0 (0%)
BRAF	1 (1.9%)	0 (0%)	1 (6.7%)	0 (0%)	0 (0%)	0 (0%)
FLT3	1 (1.9%)	0 (0%)	1 (6.7%)	0 (0%)	0 (0%)	0 (0%)
IDH1	1 (1.9%)	1 (6.7%)	0 (0%)	0 (0%)	0 (0%)	0 (0%)
KIT	1 (1.9%)	0 (0%)	0 (0%)	1 (16.7%)	0 (0%)	0 (0%)
NPM1	1 (1.9%)	1 (6.7%)	0 (0%)	0 (0%)	0 (0%)	0 (0%)
PTPN11	1 (1.9%)	1 (6.7%)	0 (0%)	0 (0%)	0 (0%)	0 (0%)
SH2B3	1 (1.9%)	1 (6.7%)	0 (0%)	0 (0%)	0 (0%)	0 (0%)

Abbreviations: MDS/MPN, myelodysplastic/myeloproliferative neoplasm; CMML, chronic myelomonocytic leukemia; MDS/MPN-N, MDS/MPN with neutrophilia; MDS/MPN-SF3B1-T, MDS/MPN with *SF3B1* mutation and thrombocytosis; MDS/MPN-NOS, MDS/MPN not otherwise specified

Table S2. Univariate and multivariate Cox regression analysis for overall survival in patients with CMML.

Variables	N (%)	Univariate analysis		Multivariate analysis	
		HR (95% CI)	*p*-value	HR (95% CI)	*p*-value
Hemoglobin					
< 10 g/dL	25 (83.3%)	4.29 (0.52–35.18)	0.175	–	
≥ 10 g/dL	5 (16.7%)	1.00			
Platelet					
< 100 × 10^9/L	17 (56.7%)	7.47 (0.92–60.71)	0.060	4.96 (0.31–79.07)	0.257
≥ 100 × 10^9/L	13 (43.3%)	1.00		1.00	
Circulating blasts					
Presence	12 (40.0%)	1.79 (0.48–6.70)	0.389	–	
Absence	18 (60.0%)	1.00			
Splenomegaly					
Presence	6 (20.0%)	3.17 (0.83–12.04)	0.091	0.47 (0.02–13.99)	0.661
Absence	24 (80.0%)	1.00		1.00	
Transfusion dependency					
Presence	12 (40.0%)	7.78 (1.55–39.23)	0.013	4.08 (0.43–39.00)	0.222
Absence	18 (60.0%)	1.00		1.00	
High-risk cytogenetics[a]					
Positive	9 (30.0%)	4.80 (0.91–25.41)	0.065	2.04 (0.18–22.61)	0.562
Negative	21 (70.0%)	1.00		1.00	
ASXL1 mutation					
Positive	15 (50.0%)	0.21 (0.04–1.01)	0.052	0.37 (0.02–5.97)	0.485
Negative	15 (50.0%)	1.00		1.00	
U2AF1 mutation					
Positive	6 (20.0%)	12.20 (1.00–148.10)	0.050	1.88 (0.05–65.40)	0.728
Negative	24 (80.0%)	1.00		1.00	

[a] -7 or del(7q), trisomy 8, and complex karyotype

Abbreviations: CMML, chronic myelomonocytic leukemia; HR, hazard ratio

Figure S1. Distribution of variant allele frequencies of *U2AF1* mutations in patients with MDS/MPN, stratified by mutation hotspot (S34 vs. Q157).

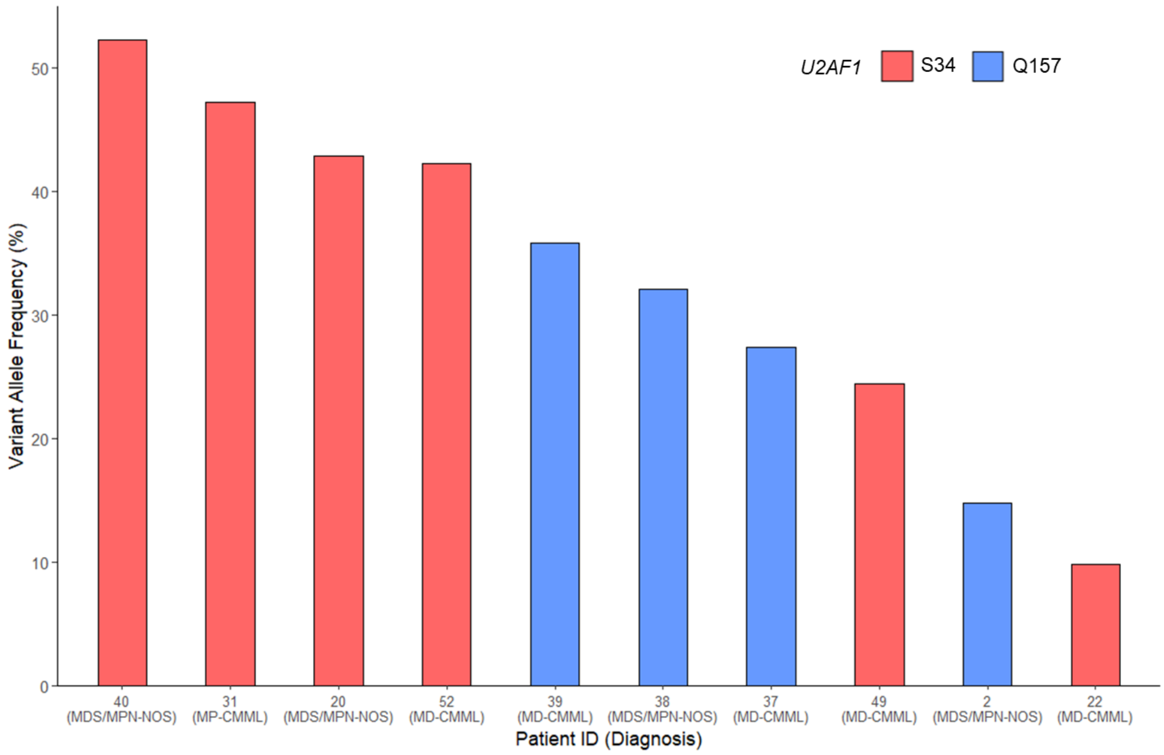

Figure S2. Forest plot of hazard ratios for 3-year overall survival in patients with CMML. Each point represents the hazard ratio with 95% confidence intervals from univariate Cox regression analysis. Variables with 95% CI upper limits exceeding 15 are truncated at the red dashed line, with the actual upper limit values provided in Supplemental Table S2.

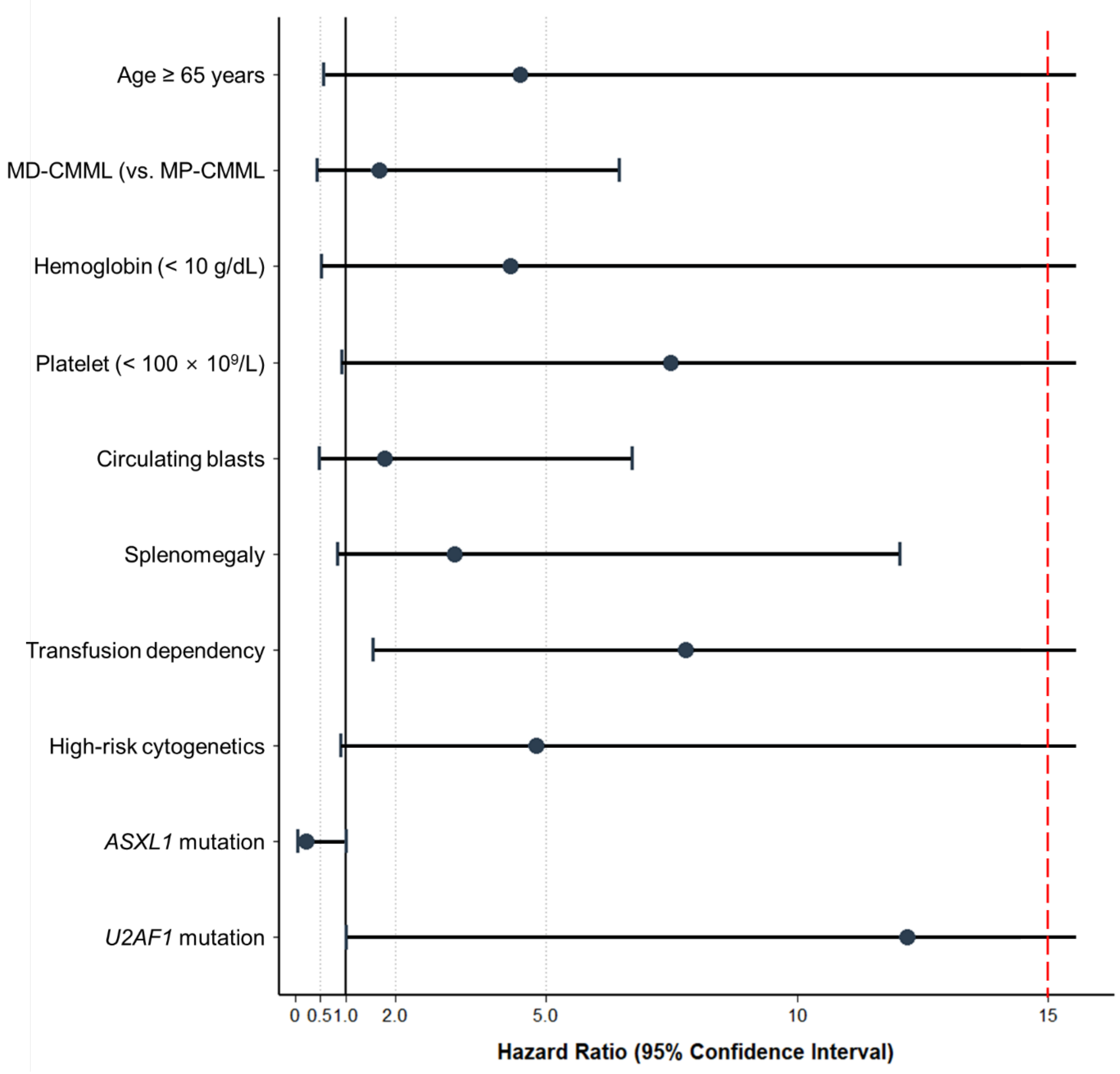

Supplement: Supplementary file 1 [file jcm-14-07074-s001.zip › jcm-3900156-supplementary.pdf]
